# Revealing the nanometric structural changes in myocardial infarction models by time-lapse intravital imaging

**DOI:** 10.3389/fbioe.2022.935415

**Published:** 2022-08-16

**Authors:** Chiung Wen Kuo, Feby Wijaya Pratiwi, Yen-Ting Liu, Di-Yen Chueh, Peilin Chen

**Affiliations:** ^1^ Research Center for Applied Science, Academia Sinica, Taipei, Taiwan; ^2^ Institute of Physics, Academia Sinica, Taipei, Taiwan

**Keywords:** real-time intravital imaging, two-photon imaging, nanometric structural change, sarcomere length, sarcomere shortening

## Abstract

In the development of bioinspired nanomaterials for therapeutic applications, it is very important to validate the design of nanomaterials in the disease models. Therefore, it is desirable to visualize the change of the cells in the diseased site at the nanoscale. Heart diseases often start with structural, morphological, and functional alterations of cardiomyocyte components at the subcellular level. Here, we developed straightforward technique for long-term real-time intravital imaging of contracting hearts without the need of cardiac pacing and complex post processing images to understand the subcellular structural and dynamic changes in the myocardial infarction model. A two-photon microscope synchronized with electrocardiogram signals was used for long-term *in vivo* imaging of a contracting heart with subcellular resolution. We found that the structural and dynamic behaviors of organelles in cardiomyocytes closely correlated with heart function. In the myocardial infarction model, sarcomere shortening decreased from ∼15% (healthy) to ∼8% (diseased) as a result of impaired cardiac function, whereas the distances between sarcomeres increased by 100 nm (from 2.11 to 2.21 μm) in the diastolic state. In addition, T-tubule system regularity analysis revealed that T-tubule structures that were initially highly organized underwent significant remodeling. Morphological remodeling and changes in dynamic activity at the subcellular level are essential to maintain heart function after infarction in a heart disease model.

## Introduction

Cardiac disorders remain a common cause of death worldwide and are likely to exhibit an increasing trend in the coming decades (Pittet and Weissleder, 2011). The cardiac function appears to be tightly regulated by the complex, tiered connection that arises from the molecular-level dynamics associated with force generation in cardiomyocytes and the geometry, stiffness, and contractility of the whole tissue; a small amount of interference in one of the processes may result in heart function failure (Sonnenblick, 1968; Kobirumaki-Shimozawa et al., 2016; van der Velden and Stienen, 2018; Yamada et al., 2020; Tobacman, 2021). For instance, errors in contractile features can lead to an overload in volume or pressure in the heart, which is commonly seen in ischemia/reperfusion (I/R) injury and myocardial infarction (MI) (van der Velden and Stienen, 2018). Additionally, impairment of one of the contractile networks may interfere with not only force generation but also the signaling processes that transmit information to cellular systems to control gene expression, protein synthesis, and protein degradation, all of which are closely related to the activities in the nucleus (Gautel, 2008; Chahine et al., 2015). Thus, an accurate measurement of the cardiac contractile system improves our present understanding of the mechanism of heart diseases.

In general, the assessment of structural and functional cardiac damage post-MI can be achieved using electrocardiogram (ECG), echocardiography, endoscopy, magnetic resonance imaging, positron emission tomography, single-photon emission computed tomography/X-ray computed tomography, or fluorescence reflectance imaging. In these approaches, spatial resolution is limited to ∼100 µm (Jung et al., 2013; Kobirumaki-Shimozawa et al., 2016). More detailed studies at the (sub) cellular level are often conducted *in vitro* using X-ray, atomic force microscopy, transmission electron microscopy, or optical microscopy (Moss and Fitzsimons, 2002). However, these *in vitro* experiments do not allow for the study of cardiomyocytes under physiological conditions.

With recent advances in fast and sensitive detectors, high-power lasers, and high computational power, it is now possible to obtain *in vivo* images of hearts with very high spatiotemporal resolution using two-photon microscopy. The development of two-photon intravital microscopy combined with various window chambers for organ imaging has proven valuable in studies of biological processes *in vivo* due to the ability to visualize deep into tissues at high resolution with low phototoxicity (Denk et al., 1990; Rubart, 2004; Kuo et al., 2019). However, due to the rapid motion of respiration and the contracting myocardium, in which the heartbeat rate of mice is approximately 6–8 Hz, it is very challenging to capture dynamic subcellular events in beating hearts (Li et al., 2013; Matsuura et al., 2018). In addition, the left ventricular (LV) position, which is located below the rib cage and sternum, cannot be easily reached by an objective lens. Consequently, cardiac studies have relied mainly on noncontracting *in vitro* preparations, or Langendorff heart preparations (Matsumoto-Ida et al., 2006; Bub et al., 2010; Botcherby et al., 2013) and heterotopic heart transplantations (Li et al., 2012), which do not represent physiological conditions.

To investigate beating hearts in mouse models, several tissue stabilization techniques for intravital imaging have been developed to reduce the considerable movement that occurs when a heart contract (Chilian and Layne, 1990; Dumont et al., 2001; Li et al., 2012; Matsuura et al., 2018). However, the required temporal or spatial resolution for subcellular imaging of cardiomyocytes has yet to be attained. Recently, cardiac pacing was introduced in the intravital microscopy system to achieve prospective cardiac gating (Lee et al., 2012; Vinegoni et al., 2013; Vinegoni et al., 2015). With image processing algorithms, such as sequential average segmented microscopy, it is possible to image individual cardiomyocytes at the cellular level (Jones et al., 2018). However, this approach requires pacing at speeds higher than that of a regular heartbeat; at these higher speeds, the heart no longer beats on its own, thus preventing long-term observation. Therefore, there is a clear need to develop long-term *in vivo* imaging techniques for beating hearts with subcellular resolutions.

In our previous experiment (Huang et al., 2021), we have shown that real-time intravital imaging could be used to visualize the lipid nanoparticle loaded monocytes in the heart disease model. By mimicking the natural recruitment of monocytes to the ischemic tissues, immune cells were used to deliver nanotherapeutics to the heart disease sites, which was validated *in vivo* by intravital imaging. However, only the movement of the monocytes were monitored. We were not sure that intravital imaging could be used to reveal the structural changes of diseased cells with subcellular resolution, which is important for the evaluation of the design of targeted nanotherapeutics. Many experiments have been carried out to investigate the morphology of organelles within cardiomyocytes in the myocardium *in vitro* or *ex vivo*, to a lesser extent, in motionless tissue, and in healthy beating hearts (Guo and Song, 2014; Kobirumaki-Shimozawa et al., 2016; Tsukamoto et al., 2016; Li et al., 2018). To the best of our knowledge, none of these experiments established systematic studies that showed a correlation between the structural and dynamic changes of these organelles in a disease state in animal models due to the weak condition of the diseased animals. Therefore, little is known about the ability of the myocardium to generate force throughout these pathological conditions (Wei et al., 2010).

In this study, we demonstrate that long-term cardiac imaging of beating hearts in mouse models at subcellular resolution can be achieved using a two-photon microscope equipped with a high-speed resonance scanner and synchronized with ECG signals in combination with a tissue stabilizer. Even though the acquisition strategy of sequence segmentation has been implemented in several imaging techniques (Lee et al., 2012; Vinegoni et al., 2013; Aguirre et al., 2014; Kuo et al., 2019), the primary difference in our system is the synchronization approach. For instance, in Weissleder’s system (Aguirre et al., 2014), the synchronization source came from the scanner, where the image acquisition rate was relatively slower than the mouse heartbeat rate. The signal generated by the scanner was recorded, processed, and used to pace the heartbeat at 8 Hz, which then allowed their recording system to capture the image. In other words, the heart was no longer beating on its own. Our system was developed to solve the abovementioned problem. Since the image acquisition speed in our system was four to five times faster than the mouse heartbeat, heartbeats were used to trigger the resonance scanner to capture the images. In this case, the heartbeat was regular, allowing long-term imaging of dynamic events at sufficient resolution, which is suitable even for disease models with conditions that make the heart weaker than those of healthy models. Thus, our technique offers several advantages over previously reported methods, including fast acquisitions, high-resolution images, and easy handling while allowing the animal to maintain a regular heartbeat (Lee et al., 2012; Vinegoni et al., 2015). Using this system, we successfully imaged the structural and dynamic activity of the contractile machinery, including T-tubulin, sarcomeres, and nuclei, of cardiomyocytes *in vivo* with sufficient spatiotemporal resolution together with clinical measures of heart function (ECG and echocardiography in both healthy and diseased mouse models.

## Materials and methods

### Imaging system setup and real-time data processing

The heart imaging system consists of an upright FVMPE-RS multimode multiphoton scanning microscope (Olympus) and a tunable mode-locked Ti: Sapphire femtosecond laser (Chameleon Vision II, Coherent) with a low-magnification air objective (XL Fluor ×5 with 0.14 numerical apertures (NA), Olympus) and a high-NA water-immersion objective (XLPlan N W MP 25 × 1.05 NA 2 mm WD, Olympus) (Tang et al., 2019; Chen et al., 2021; Huang et al., 2021).

Two personal computers (PCs) were used in this system: one was utilized to record all physiological and timing data from the ECG, while the other was used to control the microscope and acquire images. An Ethernet crossover cable was used to communicate between these two PCs. The ECG signal was acquired from a set of ECG leads, which passed through a low-noise differential amplifier (PCI-6229; National Instruments) and recorded by a DAQ (PCI-6229) at a 10-kHz sampling rate. We followed previous works in ECG denoising strategies, using discrete wavelet transformation (DWT) to remove spurious signals and trends (Mali et al., 2011). Wavelet decomposition was performed in a specified rolling window of 1,000 data points (equivalent to 100 ms) by decomposing the signal using db02 (Daubechies family) wavelets and the threshold using minimax (Donoho and Johnstone, 1998). When the QRS complex appeared in the window, the processed signal peaked around the R-wave. When the R-wave was identified, the program yielded a trigger request to the digital output, which later generated a pulse (pulse train frequency 25 Hz, duty cycle 25%) along the trigger line at a specified delay, prompting the microscope system to acquire a scan line; thus, the acquisition was completed at a given phase of the contraction cycle. The DWT was used to define the location of the QRS complexes and the start of the R waves. The timing signals were recorded using a program written in LabVIEW (National Instruments).

### Tissue staining

Membrane-specific fluorescent dye di-2-anepeq and nuclear dye Hoechst 33,342 were used to label the cardiomyocytes (all were acquired from Invitrogen). Thirty microliters of di-2-anepeq stock solution (5 mM) and 30 µl of Hoechst were administered *via* the tail vein 5–10 min before imaging.

### Surgical preparation for imaging

All experimental animal protocols and procedures were approved by the Institutional Animal Care and Use Committee of the Academia Sinica (Protocol number 19-06–1,325). Following the thoracotomy incision in the fourth left intercostal space, retractors, sutures, or hemostatic clamps were inserted in the upper and lower ribs of the incision area; then, the mediastinal window was gently opened to expose the anterior and lateral aspects of the LV. The mouse was covered with a surgical blanket that only left the mediastinal window open. Then, the mouse was transferred to the microscope stage for tissue stabilizer installation and subsequent imaging. Following the procedure, the stabilizer was positioned close to the heart surface by adjusting the position of the stabilizer arm. A drop of buffer saline was applied on the surface of the heart to prevent the tissue from drying. The stabilizer was gently placed in contact with the beating heart and carefully adjusted by controlling the custom-made micromanipulator.

### Image acquisition

During the acquisition process, the images were recorded in FLUOVIEW FVMPE-RS (Olympus software), and the timing waveforms were obtained by a LabVIEW program as described earlier. After the stabilizer was positioned on the heart, images were first captured with low magnification to examine the broad view of the heart. To observe the sarcomere and nucleus, di-2-anepeq and Hoechst were excited at a 980-nm wavelength, and fluorescence was detected using a bandpass optical filter between 410–455 nm and 575–645 nm for Hoechst and di-2-anepeq, respectively. Then, images were acquired to determine regions of interest (ROIs). For high-speed imaging, the microscope was operated in resonance mode at 30 Hz with a scanning size of 512 × 512 pixels or at 100 Hz with a scanning size of 512 × 140 pixels, with a spatial resolution of 0.198 µm/pixel. When the ROI was determined, the R-peak of the ECG signal was used as the source of synchronization to ensure that each image was recorded during the same phase of contraction cycles. Images were processed using Imaris software (Oxford Instruments).

### Quantification and statistical analysis

All image data were first processed with Imaris software, where the brightness and contrast were linearly adjusted. To quantitatively measure the sarcomere length (SL), the lines across the sarcomere structures were drawn. The distance between two sarcomeres (SL) was determined by dividing the entire structure length by the sarcomere number. The SL shortening (ΔSL) and SL shortening percentage were calculated by the following equation.

$$\Delta SL=SL_{diastole}-SL_{systole}$$ (1)

$$\%\;\Delta SL=\frac{SL_{diastole}-SL_{systole}}{SL_{diastole}}x100\%$$ (2)

Moreover, a plugin in ImageJ, TTorg (T-tubule system regularity analysis) was used to quantitatively analyze the T-tubule structures. The algorithms used to calculate the transverse organization level of a TT network are based on calculating the peak amplitude in the Fourier spectrum of the image at the TT frequency. The transverse organization indicator, TT_power_ value, was determined; a higher value indicates a uniformly transverse organized structure. Results were reported as mean ± stdv. Statistical analysis of the data was performed using one-way ANOVA Tukey post hoc test provided by Origin 2018. Statistical significance was set at *p* < 0.05.

## Results and discussion

### Strategy for imaging a beating heart in a mouse model

**FIGURE 1:**
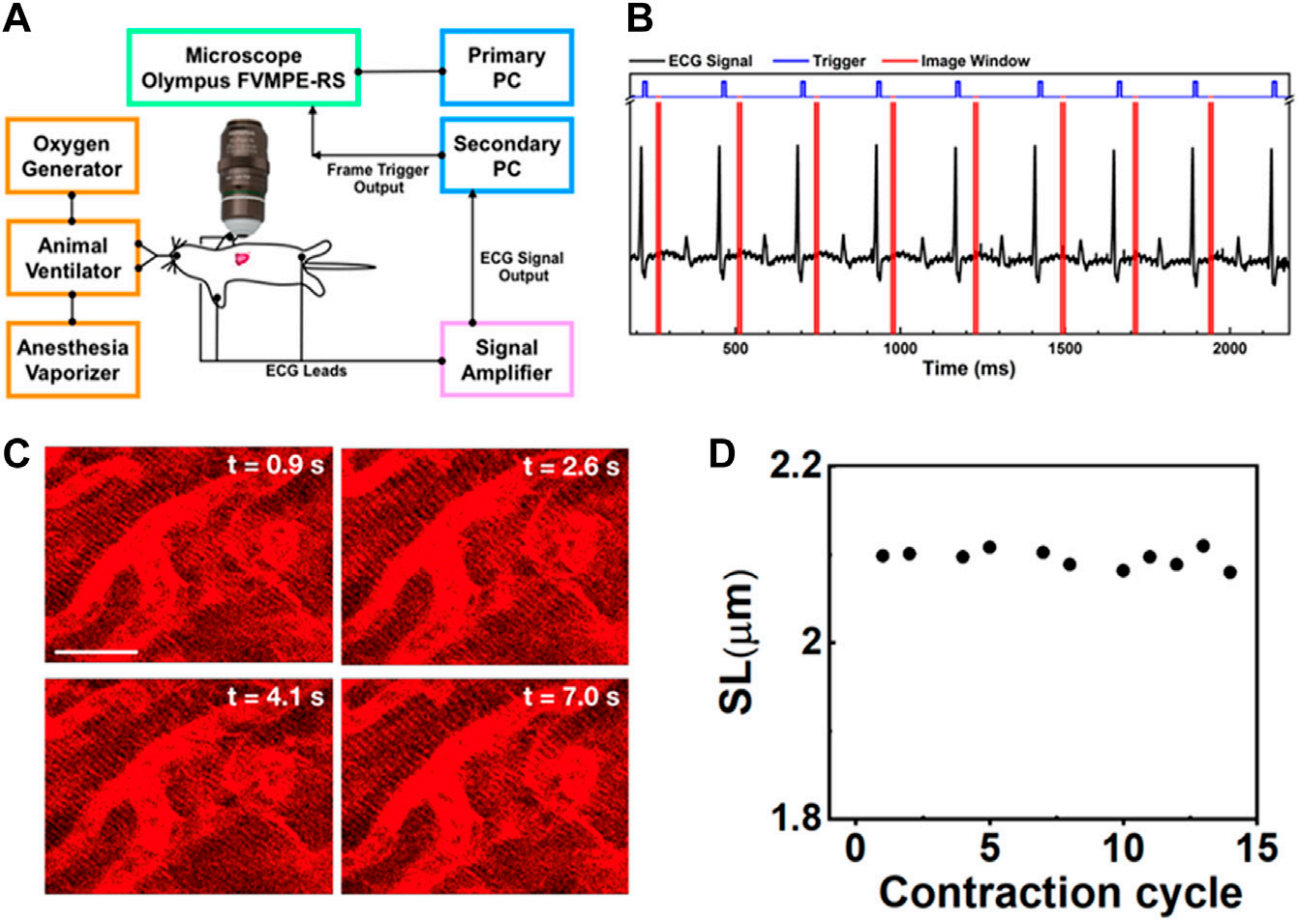
**(A)** Schematic intravital cardiac imaging system. The R waves of ECG signals from the animal are used to trigger the two-photon microscope allowing imaging of the heart at a specific phase during each contraction cycle. **(B)** The timing diagram used for ECG synchronization of image acquisition—black: ECG signal from the mouse, blue: trigger prompting image acquisition, red: acquisition time window. By tuning the delay time between the R-wave and image acquisition, different phases during the contraction cycle can be imaged. **(C)** Two-photon images of cardiomyocytes in the beating heart synchronized with the ECG signal at the same phase of four consecutive contraction cycles (label as time points). The variation in each image is very small indicating that the system is capable of recording clear and stable images on a beating heart without image reconstruction. Scale bar: 20 µm. **(D)** The SL was measured from the images of cardiomyocytes taken at a specific phase in different contraction cycles.

Among the major organs, the imaging of a beating heart remains challenging. Typically, the heartbeat rate of mice is approximately 400–500 times per minute or 6–8 Hz. According to the Nyquist-Shannon sampling theorem in bandwidth signals, a minimum image acquisition time should be at least twice as fast as the beating rate to avoid distortion and blurring and to restore the original signal if the motion is reproducible (Li et al., 2018; Ravanshad and Rezaee-Dehsorkh, 2020). However, the heart images recorded in a mouse by a conventional two-photon microscope are blurry due to rapid and nonuniform heart motion (Supplementary Movie S1), of which movement occurs at speeds up to 19.9 mm/s under physiological conditions and increases to 47.8 mm/s when animals are on ventilation (Stohr et al., 2018; Kavanagh and Kalia, 2019). To overcome motion-induced artifacts, a strategy combining synchronization, stabilization, and cardiac surgery was introduced to suppress artifacts in imaging contracting hearts in this experiment, as shown in Figure 1A.

To improve the imaging quality, we used a custom-made tissue stabilizer consisting of a flat polymer ring with a glass coverslip (d = 1 cm) at the bottom attached to a 3 cm long rigorous metal rod (Supplementary Figure S1). This tissue stabilizer was mounted on a micromanipulator stage, where the stabilizer could be moved in the x, y, and z directions. Throughout the entire acquisition period, the cover glass ring kept the lens immersed in a saline solution that matched the refraction index of the imaging objective. Our setup prevented the objective lens from directly touching the heart. Thus, it allowed the heart to return to its original position over time in a reproducible manner. The only remaining pressure came from the heart itself when it contracted toward the ring, similar to the heart beating toward the chest wall. As a result, blood flow was normal, with minimal perturbations. In contrast to other stabilizing techniques involving bonding the heart with mechanical stabilizers, such as sutures, restraining coverslips, or suction-based devices, (Li et al., 2012; Matsuura et al., 2018), which suppress excessive motion in its entirety as much as possible, the purpose of using a mechanical tissue stabilizer was to reduce excessive motion artifacts. A stabilizer can sufficiently reduce the displacement of the heart up to a few micrometers and allow the heart to return to its original position over time, therefore improving the motion reproducibility across the cardiac phase. This strategy is sufficient to enable the acquisition of an image of a beating heart with low resolution, including aspects of the heart such as vasculature, microvasculature, blood flow, and cell recruitment (Jones et al., 2018).

In this experiment, two-photon microscopy equipped with a resonance scanner was used, which allowed cardiomyocytes to be imaged at a frame rate of 30 Hz with 512 × 512 pixels (Supplementary Movie S2A). Even though the imaging acquisition rate using a resonance scanner was sufficient to capture cardiomyocytes without any distortion when using a tissue stabilizer, the relative positions of the cells carried over from one frame to the next. Therefore, image alignment is necessary to reduce motion-induced artifacts. Moreover, the heartbeats not only in the lateral direction but also in the axial direction. As a result, an image sequence at a fixed objective lens position contained severe motion-induced blurriness or did not allow proper acquisition of selected cells, therefore restricting the ability to capture sequential heartbeat dynamics (Taylor, 2014). The imaging speed can be further increased to 100 Hz by reducing the scanning area to 512 × 140 pixels (Supplementary Movie S2B). At 100 Hz, we could capture the periodic variations in sarcomeres. However, the reduced dwell time at this high speed can severely degrade the quality of the image, leading to rapid bleaching of the dyes; these occurrences result in lower total image signal-to-noise ratios, which is not suitable for acquiring long-term measurements (Kobirumaki-Shimozawa et al., 2016).

To reduce motion-induced blur, we synchronized the contraction cycle with the scanner of a two-photon microscope, where an ECG system feeds live signals from the cardiac cycle to provide proper triggers for data acquisition (Figure 1A). The timing diagrams presented in Figure 1B, Supplementary Figure S2 show the synchronization process of our imaging system. The R waves from the mice’s ECG signal (black) triggered image acquisition in the system (blue), while the red color represents the imaging acquisition window. The R peak was used as the source of synchronization to ensure that each image was recorded at the same phase of the contraction cycle. In this way, the heart position would have the same focal point within the imaged organ at different time points so that the image frame would have high quality. This setup also ensures that a high-quality frame is acquired at the accurate phase in the heart’s cycle rather than requiring continuous high-frame-rate acquisition to assure that one of the frames recorded is at approximately the correct position of the heart’s cycle. Reconstructing images at several different phases provides cellular and dynamic information about the entire heartbeat cycle.

Supplementary Movies S3A–D show the dynamic images at different delay times, which correspond to different phases of the cardiac cycle. Figure 1C shows representative heart images captured at four consecutive contraction cycles (label as time points) at the same phase with respect to the R-wave of ECG signals. Each image is stable from one frame to the other without distortion, blur, and significant shifting of sarcomere structures. Thus, the sarcomere length (SL) from these captured images could be reliably measured at each delay time (Figure 1D, Supplementary Figure S3). Even though the tissue stabilizer alone was able to confine the movement of the heart, the residual motion artifacts still strongly affected the reconstructed images (Supplementary Figure S4A). In contrast, when the scanner was synchronized with the R-wave of the ECG signal, those motion-induced artifacts could be removed from the images (Supplementary Figure S4B), preventing the loss of image resolution from the motion artifact. With the synchronization approach, high-resolution images could be obtained.

### Nanometric imaging of sarcomeres in the beating hearts of healthy mice

**FIGURE 2:**
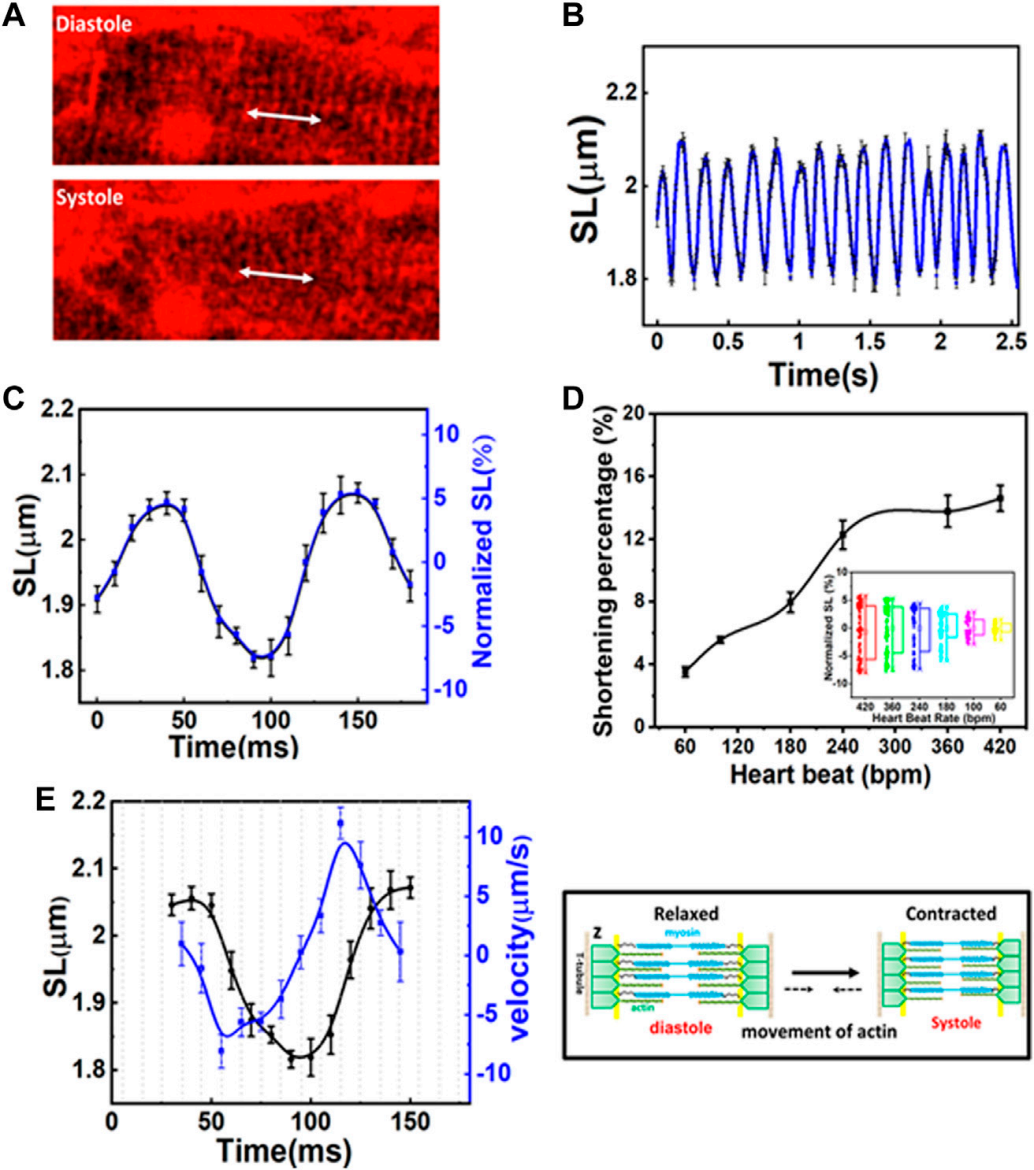
**(A)** Two-photon images of the same cardiomyocyte at a different phase of the contraction cycle. **(B)** Variation in sarcomere length (SL) over time in several contraction cycles. **(C)** The original and normalized SLs at different phases in the contraction cycle. The SLs are measured at a given phase (at a delay time with respect to R-wave) in a contraction cycle and averaged over several contraction cycles. **(D)** The shortening percentage (%ΔSL) and normalized SL variations were measured at different heartbeat rates. **(E)** The correlation between SL (left Y axis) and shortening velocity (right Y axis) in one cardiac cycle (*N* = 3, mean ± standard deviation). A positive velocity represents sarcomere lengthening whereas negative velocity indicates sarcomere shortening.

The capability to record structural changes within a cell in the beating heart is crucial to the understanding of functional changes in physiological and pathological conditions. To visualize the dynamic behavior of cardiomyocytes, the lipophilic potentiometric dyes di-2-anepeq and Hoechst were intravenously injected into mice. The rapid transient response of di-2-anepeq to membrane potential changes allows effective staining of the membranes, including the external membranes of cardiomyocytes and transverse tubules (Bub et al., 2010). The array of transverse-axial tubules (T-tubules) can be visualized using high magnification at different states within a contraction cycle, as shown in Figure 2A. The distance space between T-tubules, which is parallel to the z-disk, was measured and defined as the SL. From the recorded time-lapse images of the cardiomyocytes in a beating heart, we observed synchronized movement between regularly repeated sarcomere shortening and myocyte movement in a beating heart. The SL measured from individual cardiomyocyte contraction and relaxation was highly reproducible for different mice in multiple heartbeats (Figure 2B). Our results indicate that the maximum SL is approximately 2.08 µm in diastole and contracts to approximately 1.81 µm in systole (Figure 2C). The overall changes in SL (ΔSL) in the cardiomyocytes of a healthy mice beating heart during the contraction cycle are less than 300 nm. Moreover, since hemodynamic parameters (i.e., ECG) of the heartbeat were simultaneously recorded with real-time sarcomere dynamics, it allowed us to investigate the relationship between SL and its variations during contraction cycles at different heart rates (Figure 2D, Supplementary Figure S5). We observed slightly decreases of shortening percentage (%ΔSL) at a heart rate between 420 and 240 bpm (Kobirumaki-Shimozawa et al., 2016). However, %ΔSL decreased linearly over a slower heart rate of less than 240 bpm (Hanft et al., 2008; Serizawa et al., 2011).

Figure 2E shows the correlation between the time-dependent changes of SL and velocity during one cardiac cycle. The positive and negative velocities (blue curve in Figure 2E, right Y-axis) indicate that sarcomeres undergo either lengthening or shortening, respectively. Thus, velocity relates to the association and dissociation of thin and thick filaments on force-generating cross-bridge formation (Hanft et al., 2008). When ejection begins, the sliding rate of myosin in actin filaments and the number of cross-bridges dictate the degree of shortening (ΔSL). In the first 10 ms (Figure 2E, X-axis) after the diastolic phase, sarcomere shortening occurs at a maximum velocity of ∼8 µm/s (Figure 2E). As the sarcomere shortens to less than 2 µm (Figure 2E, left Y-axis), its velocity gradually decreases (i.e., duration 55–100 ms) until the sarcomere reaches ∼1.81 µm which is relatively constant for another 20 ms (systolic phase). This may be because thin filaments sliding into the opposite half of the sarcomere may interfere with one filament or disrupt bond formation in the opposite half of the sarcomere, thus reducing force development and shortening velocity (Gordon et al., 2000). Additionally, at this point, the filaments shift farther apart laterally, reducing the chances of force-generating bridge reforms along the filament. Sarcomere shortening is then followed by the relaxation phase, which initially involves a relatively slow increase in length (in the first 30 ms), cooccurring with fast stretching (∼11 µm/s), and a return to slow increase in length when reaching the diastolic peak. The first slow phase attested to the change in force in non-force-generated cross-bridge states (Stehle et al., 2002; Pasqualin et al., 2015). Shortly after that, the rapid lengthening indicates its mechanical relaxation. Overall, the average velocity between lengthening and shortening is relatively the same (Gordon et al., 2000; Stehle et al., 2002; Moss et al., 2004). Since the lengths of the thick filaments (approximately 1.5 µm) and thin filaments (1.0 µm) are constant, the I-band, which contains the titin spring and connects the thick filament to the z-band, elongates and shortens to ∼0.15 µm during the contraction cycle. This extensible region plays a prominent role in passive resistance over the working range of cardiac sarcomeres (Tonino et al., 2017; Freundt and Linke, 2019). Through a repetitive association and dissociation of cross-bridge formation, force is produced. At a heart rate of 420 bpm, sarcomeres contract more than 200 nm from their diastolic length; this contraction approximately equals 3–5 units of calcium-binding troponin (each unit ∼38.5 nm) in each thin filament (Hanft et al., 2008; Yamada et al., 2020).

### Revealing the subcellular structure changes in the MI model

In mouse models of heart diseases, such as MI or I/R models, heart function is impaired. Therefore, a gentler technique is required for long-term *in vivo* imaging. As mentioned above, synchronization between physiological signals and data acquisition allows imaging to be performed at a specific phase during the contraction cycle with minimized motion-dependent artifacts and photodamage; therefore, this approach is suitable for long-term observation even for the MI mouse model. The effects of MI were studied 1 day after the induction of infarction of the LV wall *via* left anterior descending (LAD) ligation. For a control experiment, a sham model was also prepared (Lindsey et al., 2018). The heart function of the mice (both control and MI model) was examined using ultrasound imaging in M-mode (Supplementary Figure S6A). The echo data showed that the ejection fraction (EF, %) and the fraction shortening (FS, %) of the heart significantly decreased at 1-day post-MI, from 72.01 ± 6.29% to 43.26 ± 3.85% and from 35.93 ± 4.96% to 18.03 ± 1.99%, respectively, corresponding to reduced heart function. Two-photon imaging with ECG synchronization was performed to observe changes in cellular morphology when heart function was compromised. ECG signals also provided real-time heart function data for both groups of mice during the imaging process (Supplementary Figure S6B). In contrast to sham mice, post-MI ECG signals showed the development of pathological Q waves, decreased R-wave amplitude, and repolarization abnormalities, which was indicated by the depression of the JT/T segment described in previous reports (Merentie et al., 2015). Moreover, the R-amplitude, which represents depolarization during ventricular action potentials (Kaese and Verheule, 2012), decreased (up to 0.3 mV) with a reduction in the heart’s pumping functions.

**FIGURE 3:**
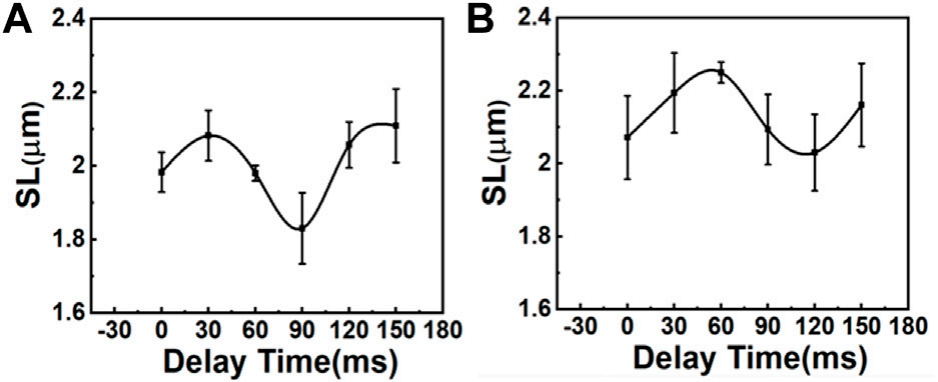
Comparison of SL variations during the contraction cycle between **(A)** sham mice and **(B)** mice with MI (*N* = 3, mean ± standard deviation). The variation of SL is larger in sham mice than in mice with MI.

In this experiment, the R-wave from the ECG signal was used to trigger the resonance scanner at various delays to access different phases of the cardiac cycles. With this approach, we were able to measure the ΔSL in the cardiomyocytes of the control (sham) and MI mice. Figure 3 depicts the average distance between the sarcomeres at different time delays with respect to the peak of the R-wave, where a time delay with an increment of 30 ms was used to probe different phases in the cardiac cycles (Supplementary Figure S6C). In the sham model, the SL was shortened from ∼2.11 to ∼1.83 μm (Figure 3A), which was a change of more than 100 nm in both directions (∼15% of the initial length). In contrast, the SL in post-MI mice (Figure 3B) increased, but the ΔSL between diastole (∼2.21 µm) and systole (∼2.07 µm) decreased to approximately 50 nm in both directions (∼8% of the initial length). Thus, our results (Table 1 and Supplementary Figure S7) support previous studies that have reported a positive correlation between the SL shortening ratio and heart function (EF or FS), and indeed, this relationship is similar to ventricular myocytes isolated from different animals (regardless of the animal’s heart size) (Sonnenblick, 1968; Cheng et al., 1995; Bers et al., 2003).

**TABLE 1:** Correlation between the sarcomere shortening percentage (% ΔSL) and heart function (EF and FS).

	ΔSL (%)	EF (%)	FS (%)
Sham	15.62 ± 0.73	72.01 ± 6.29	35.93 ± 4.96
MI	8.39 ± 1.85	43.26 ± 3.85	18.03 ± 1.99

**FIGURE 4:**
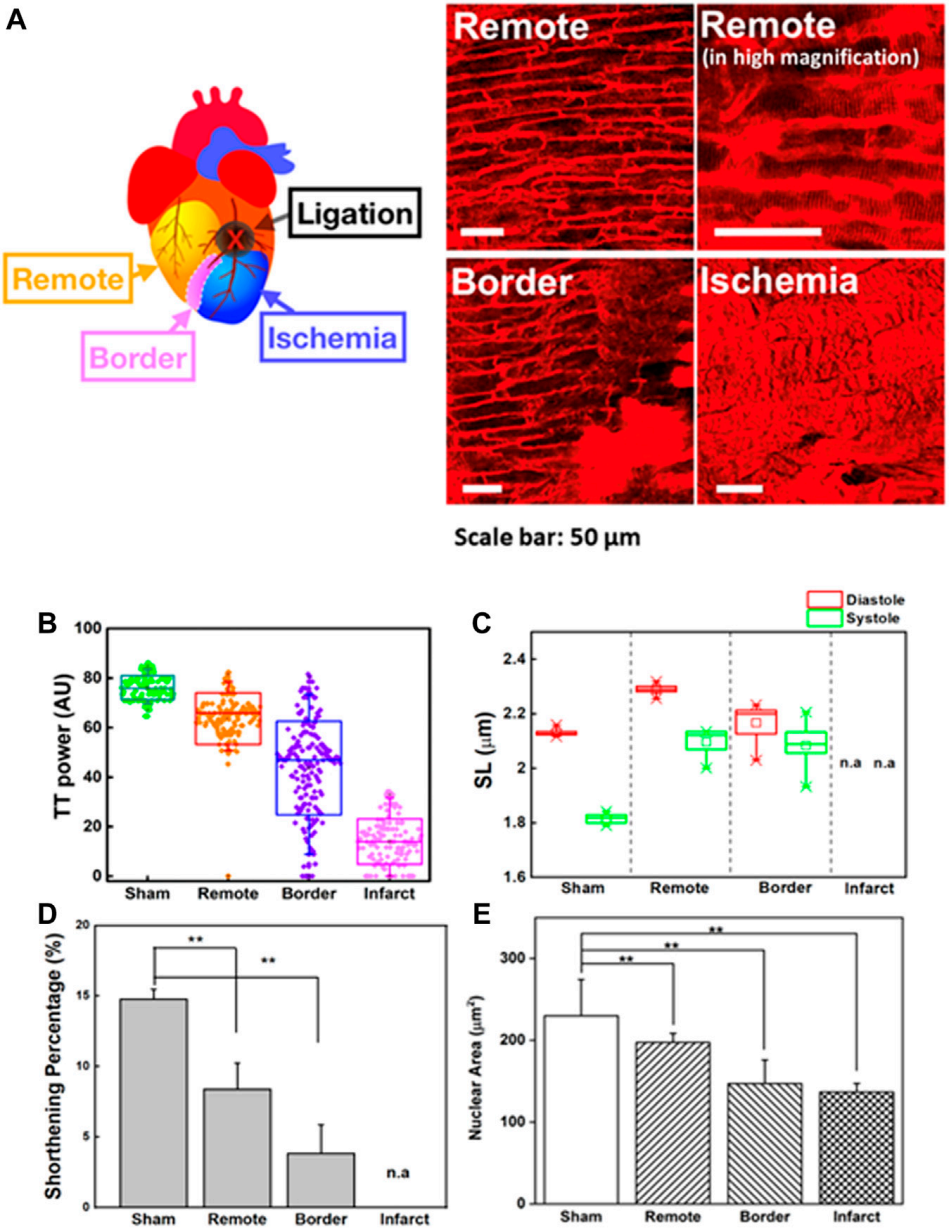
**(A)** Two-photon images of cardiomyocytes in a different region of the heart in an MI model. Remote area is far away from the point of ligation where the cardiomyocytes are normal. Higher magnification image of the same remote area is provided to reveal the fine structures of sarcomeres. Scale bar: 50 µm. **(B)** T-tubule system regularity analysis of the heart in sham and different regions of MI models (remote, border, and infarct). Higher TT_power_ indicates ordered organization of T-tubule system, **(C)** Sarcomere length (SL) variation in the systolic and diastolic states of the sham and different regions of MI models (remote, border, and infarct). **(D)** The sarcomere shortening percentage ( % ΔSL ) of the heart in the sham and in different regions MI models (remote, border, and infarct). **(E)** The nuclear area in different regions of the heart in the MI model (remote, border, and infarct) compared to the sham model (*N* = 3, mean ± standard deviation).

The heart-pumping process is associated with precise inter-organelle communication in cardiomyocytes, such as the signal transmission of the sarcolemma with the sarcoplasmic reticulum during EC coupling (Bers, 2002; Yamada et al., 2020). In this process, the transverse tubular system facilitates rapid and synchronized transmission of the membrane action potential. Figure 4A depicts the morphological changes in T-tubules and sarcomeres in different regions of the heart post-MI (remote, border, and infarct areas). To quantitatively analyze the T-tubule organization, we used a plugin in ImageJ, TTorg (T-tubule system regularity analysis). The TT_power_ that defines the organization level and the regularity of T-tubules in cardiomyocytes were determined in the sham, and different regions of MI mice model (Figure 4B). A highly organized T-tubule structure is shown in sham (TT_power_ ∼76%) and remote area (TT_power_ ∼64%) of MI mice model. However, due to mechanical stress during MI, many cells undergo hypoxia and, finally, necrosis and apoptosis; these occurrences are depicted by a blurring of striated patterns and depletion/disorganization of the T-tubule array in the sarcomere system which are observed in border (TT_power_ ∼44%) and infarct regions shown a lower value indicated poor transverse organized structure and infarct region (TT_power_ ≤ 20%) (Yue et al., 1998; Zhang et al., 2010).

Here, contractility in a different region of the heart post-MI was observed (Figures 4C,D). To compensate for the loss of contractile activity and to preserve contractility function, sarcomere lengthening and reorganization in the remote region was ∼2.21 ± 0.01 µm in the diastolic state (sham = 2.11 ± 0.1 µm; border = 2.17 ± 0.02 µm) (Figure 4C) (Rusu et al., 2019). However, continuous stress and stretching during adaptation could alter the structural properties of titin, which contributes to the interruption of the sarcomere-generated force production mechanism as well as its implication in the dilated heart (Stohr et al., 2018; Freundt and Linke, 2019). Throughout MI progression, we observed that continuous infarct expansion induces the deformation of cardiomyocytes in the border zone and remote myocardium, eventually altering sarcomere shortening (ΔSL). The percentage ΔSL at the border (3.83 ± 2.04%) was lower than that at the remote sites (8.39 ± 1.85%) and sham group (14.76 ± 0.73%) (Figure 4D). We found that T-tubule network organization was closely related to shortening percentage of sarcomere (ΔSL) (Supplementary Figure S8A). The highly organized T-tubule network is critical for normal electrical excitation-contraction (EC) coupling and cardiac function (Guo et al., 2013). Thus, it allows synchronized contraction among the many contractile units of entire cardiomyocytes to generate highly efficient heart power (Abhilash et al., 2014; Awasthi et al., 2016). However, the alteration in T-tubule organization post-MI (TT_power_ ≤ 20%) may disturb the communication between dihydropyridine and ryanodine receptor channels, therefore causing changes in the amount of Ca^2+^ administered to the contractile machinery and Ca^2+^ handling dysfunction in cardiomyocytes, resulting in disturbed cardiomyocyte contraction and relaxation (Guo et al., 2013). Moreover, it is believed that T-tubule remodeling begins much earlier in disease progression, even before it is detectable through echocardiography or before LV systolic dysfunction, and it undergoes gradual deterioration from compensated hypertrophy toward the heart failure (Freundt and Linke, 2019).

The molecular architecture of striated cardiomyocytes includes intermediate filaments interwoven in the Z-lines of sarcomeres, which build a mechanotransduction channel connecting the shape of cardiomyocytes and nuclei (Bloom, 1970; Lammerding et al., 2004; Lee et al., 2017). The alteration of cellular mechanical force balances post-MI results in an integrated change in the cellular, cytoskeletal, and nuclear shape dimensions (Lammerding et al., 2004; Dahl et al., 2008; Gupta et al., 2010; Li et al., 2019). Despite the importance of the nucleus to the function of cardiomyocytes, there are limited studies exploring nuclear structures and their roles in heart diseases compared to the structure and roles of other cardiomyocyte components (Chen et al., 2012; Chahine et al., 2015). Supplementary Movies S5A,B revealed that the nucleus was highly aligned along with the cardiomyocyte orientation and underwent conformational variations during the contraction-relaxation cycle. In the diastolic state, the shape of the nucleus is smoothly oval, but for every incremental decrease in SL, the nucleus shape slowly compresses longitudinally until it reaches the shape observed in the systolic state (Supplementary Movie S5C). Moreover, in the systolic phase, wrinkling of the nuclear envelope could be detected, indicating that intracellular forces deformed the nucleus of spatially confined cardiomyocytes (Bloom, 1970). The nuclei were restored to their original shapes and size during the diastolic phase, indicating that the nucleus possesses elasticity properties in the normal range of pressures induced by heart contraction. However, the elasticity is no longer preserved when a sarcomere is severely damaged, as it would be in the infarct region (Supplementary Movies S6A,B). The morphological changes in sarcomeres induce the remodeling of the nuclei as well.

The dynamic morphological change of the nucleus can be quantified by examining nuclear lengths, widths, and areas during these contraction cycles (Figure 4E). The nuclear area of the infarct and border regions (border = 146.98 ± 28.90 µm^2^; infarct = 136.71 ± 10.52 µm^2^) decreased compared to that of the sham group (sham = 229.99 ± 44.28 µm^2^). Indeed, the reduced area of the nucleus was positively correlated with irregularity and disorganized T-tubulin array (Supplementary Figure S8B). In contrast, we observed nuclear enlargement in a remote area of the post-MI heart (197.25 ± 11.09 µm^2^) compared to the border and infarct, which may indicate that changes in cellular metabolism and/or signaling pathways are an adaptive process to accommodate the loss of viable cardiomyocytes (Sims et al., 1992; Webster et al., 2009; Chahine et al., 2015). These results agreed with previous *in vitro* reports where nuclear membrane deformation was generated by sarcomere shortening via mechanical connections (Houben et al., 2007). The alteration of cellular mechanical force balances post-MI results in an integrated change in the cellular, cytoskeletal, and nuclear shape dimensions (Lammerding et al., 2004; Dahl et al., 2008; Gupta et al., 2010).

## Conclusion

A relatively simple design and gentle technique of two-photon imaging can have broad applicability for real-time study of the dynamic change in myocardial tissues *in vivo* and complex disease models, such as MI, in which the condition of the heart is weaker than that in healthy animals. Our results show that a highly organized T-tubule structure is critical for normal EC coupling and cardiac function. Thus, determination of SL and changes in SL (ΔSL %) during contraction, which correlate with the physiological condition of cardiomyocytes, are essential for understanding the physiology of heartbeats within the whole heart. Overall, the high synergy of geometry and the dynamic behavior of contractile units in the same ventricular myocyte and among millions of working ventricular myocytes allows the heart muscle to produce maximal contraction efficiency. However, a small interference in one of the cardiomyocyte components can result in failure of the heart’s function. Finally, monitoring the dynamic cellular process at subcellular resolution *in vivo* can provide new insights into the design of nanomaterials for targeting to the diseased sites of cardiac disorders.

## Data Availability

The original contributions presented in the study are included in the article/Supplementary Material, further inquiries can be directed to the corresponding author.
