# Supplementary material for: Revealing the nanometric structural changes in myocardial infarction models by time-lapse intravital imaging
Source: Front Bioeng Biotechnol. 2022 Aug 16;10:935415. doi: 10.3389/fbioe.2022.935415 (PMC9424828; doi:10.3389/fbioe.2022.935415)
Supplement: Supplementary file 6 [file DataSheet1.pdf]

Supplementary Material

Material and Methods

Surgical Preparation

First, deep anesthesia was induced by putting mice in a chamber filled with a flow of enriched oxygen (92–95%) containing 1.5–2% vol/vol isoflurane at a rate of 1.5–2 l/min. After that, the anesthetized mice were transferred to warming pads with a plate board on top. The temperature of the warming pads was held at 37 °C using a temperature-controlled heat plate (Kent Scientific PhysioSuite) to maintain the animals' body temperature during the surgery. A nose cone was quickly placed on the noses of the mice to administer oxygen-containing isoflurane. For ECG monitoring, the paws of the mice were taped to the plate board, and the ECG leads were subcutaneously tunneled into their legs. The grounding, positive, and negative leads were positioned on the right hind leg, right front leg, and left front leg, respectively; this setup was equivalent to the "left arm–right arm" ECG configuration. Subsequently, 0.1 mg/kg buprenorphine was injected before starting the surgery. Before performing the thoracotomy, the intubation procedure was carried out by inserting a 22-G angiocath into the trachea (as an endotracheal tube) via the vocal cords. Then, the angiocath was connected to ventilator tubing (RWD Life Science Co. Lbd Model: RW D405). The hair on the left chest area (thoracic cavity) was removed, and the area was cleaned using 70% ethanol and topical betadine. A cut of approximately 20–35 mm was made in the chest skin from the left axilla along the fourth intercostal to the left sternal border. The tissue and superficial muscular layer were carefully cut using cautery to expose the rib without injuring the internal thoracic artery. Then, a thoracotomy incision was made in the fourth left intercostal space.

MI model and sham

The six- to eight-week-old C57BL/6 mice that were for this experiment were provided by the preclinical core facility for cardiovascular and stroke animal models in regenerative medicine, Academia Sinica. Each mouse was ventilated at a tidal volume of 0.2 ml at 120 breaths/min, and enriched oxygen (92-95%) containing 1.5–2% (vol/vol) isoflurane flowed through a nose cone that had been placed on the mouse. After thoracotomy, the left coronary artery was intramurally tied with an 8-0 Prolene suture to induce myocardial ischemia (MI), while for the sham model, a similar process was carried out without ligation; then, the Prolene suture was removed [39]. After the treatment, the animals were kept for recovery before the imaging process. Each MI was confirmed by echocardiographic visualization.

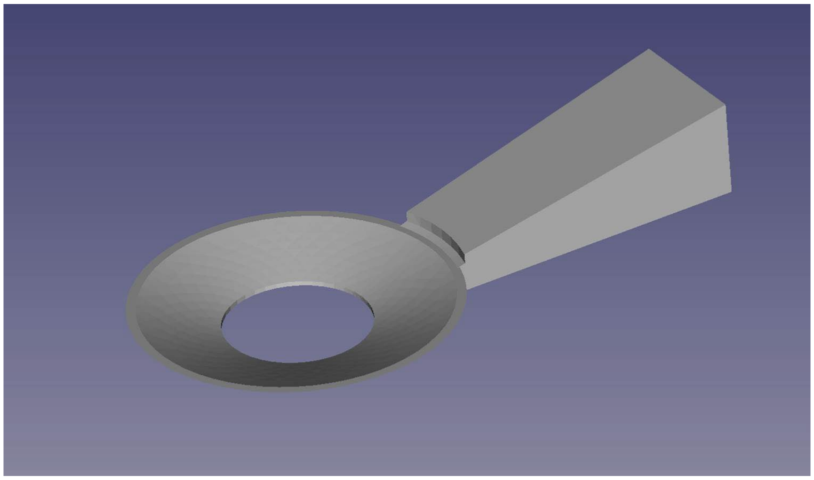

Supplementary Figure S1. The three-dimensional view of the tissue stabilizer, which is consisting of a flat polymer ring with a glass coverslip (d = 1 cm) at the bottom attached to a 3 cm long rigorous metal rod.

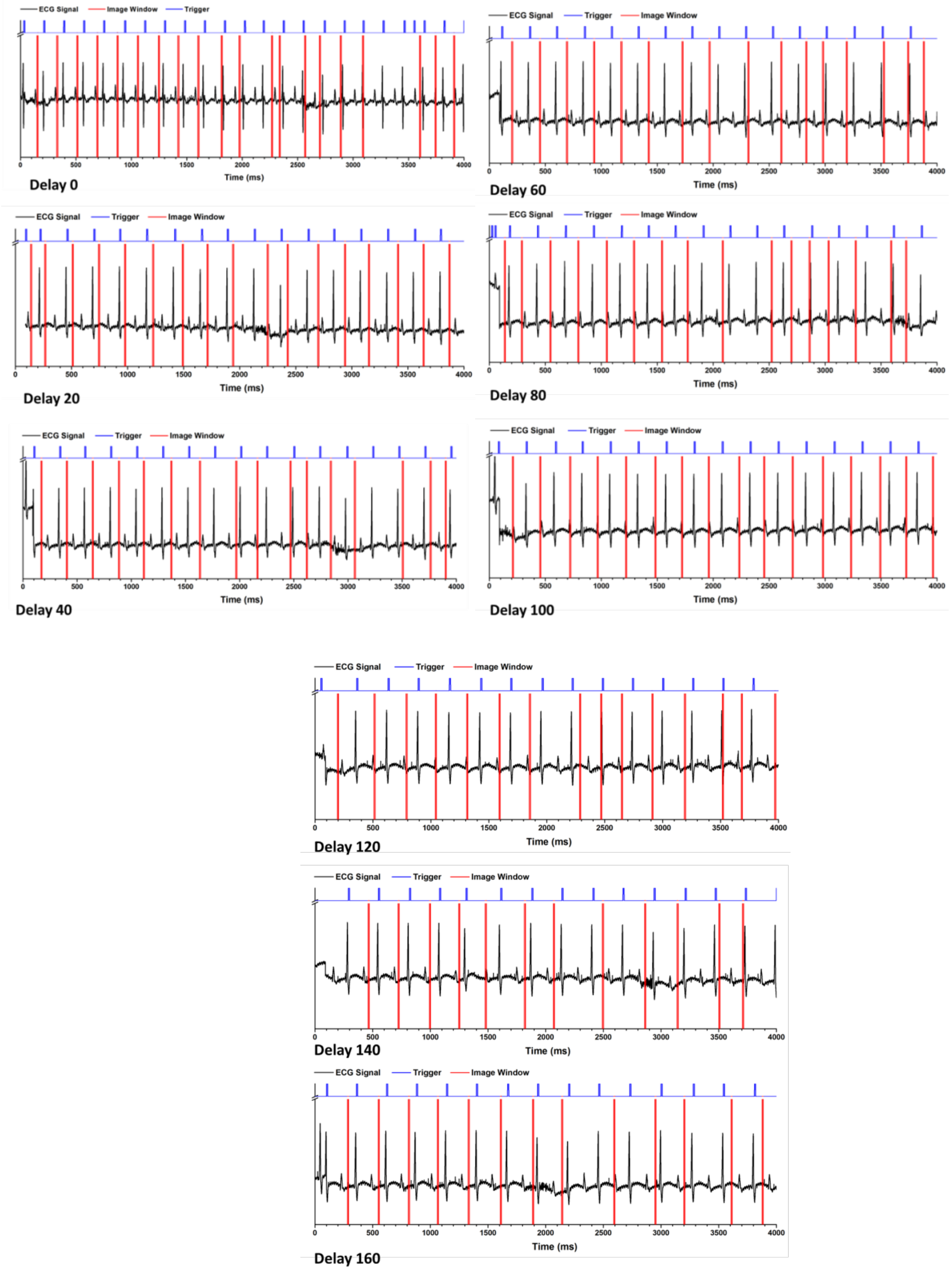

Supplementary Figure S2 The electrocardiograms (black), the trigger signals (blue), and the imaging window (red) at various delay times. The main peak (R-wave) triggers the scanner and the image is acquired at a time delay with respect to R-wave.

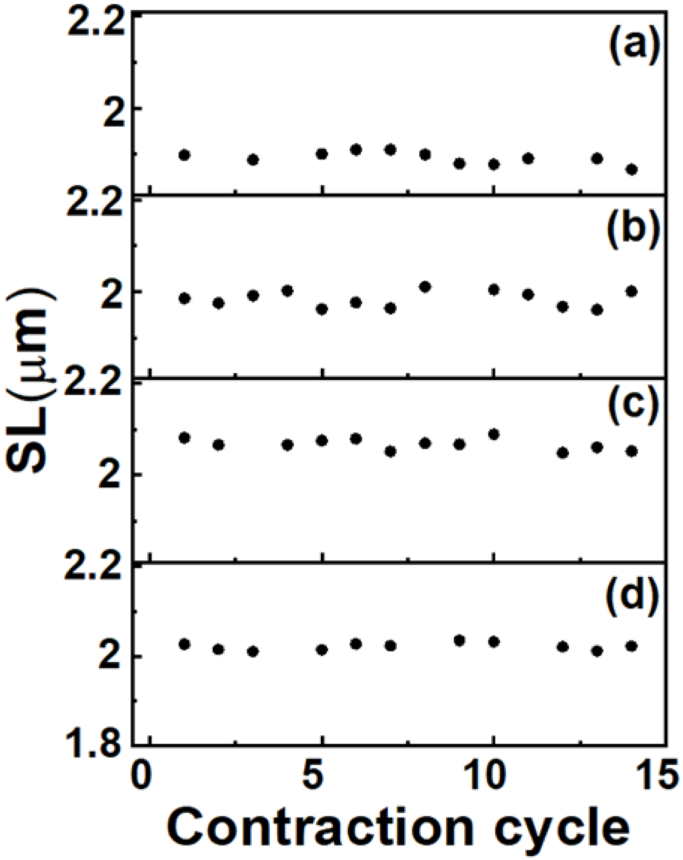

Supplementary Figure S3. The sarcomere length of control mice measured at different time delays (a) 90, (b) 60, (c) 30, and (d) 0 ms measured from Movie S3 a-d

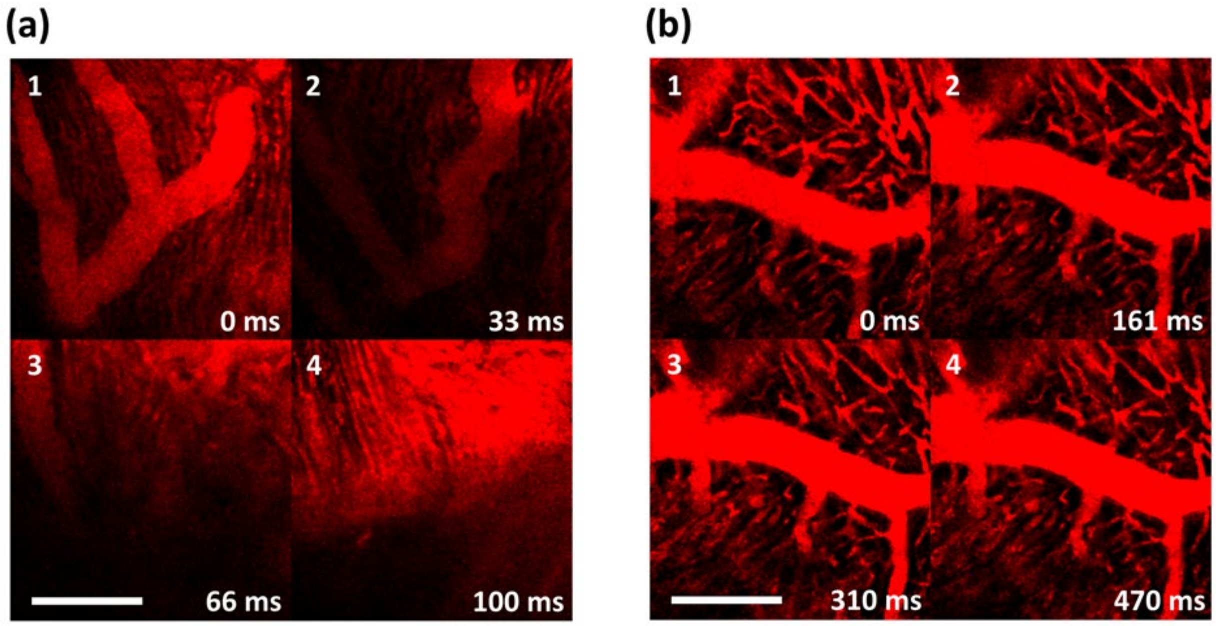

Supplementary Figure S4. The image of a beating heart with (a) the presence of only the motion compensation stabilizer, (b) the presence of both motion compensation stabilizer and ECG synchronization. Scale bar 200 µm

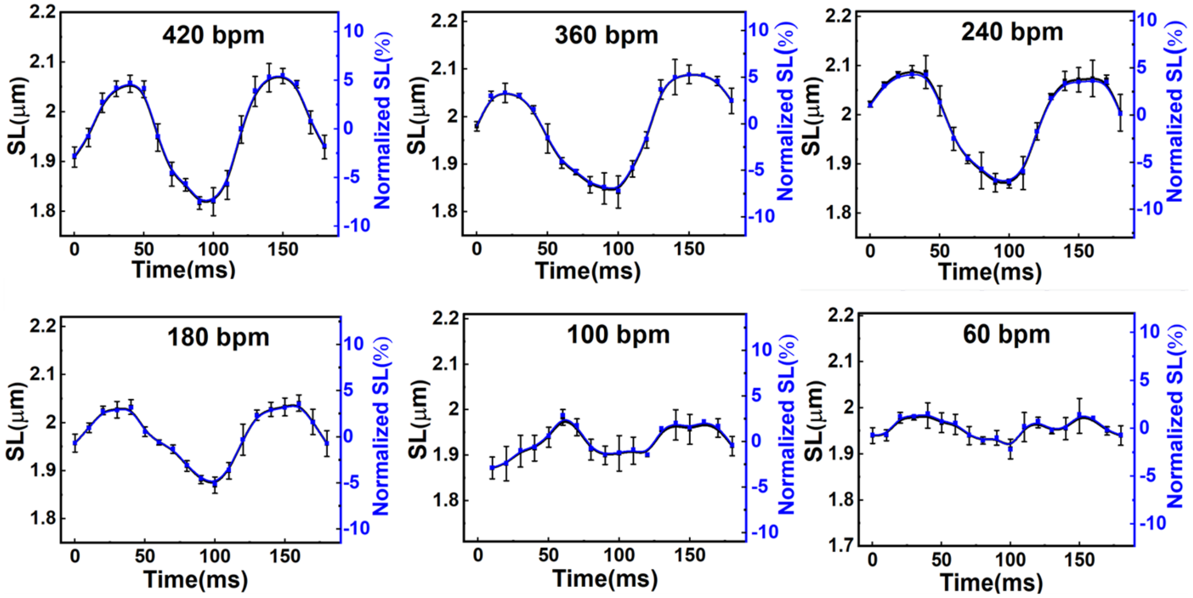

Supplementary Figure S5. The sarcomere length was measured at different phases of the contraction cycle for mice with different heartbeat rates.

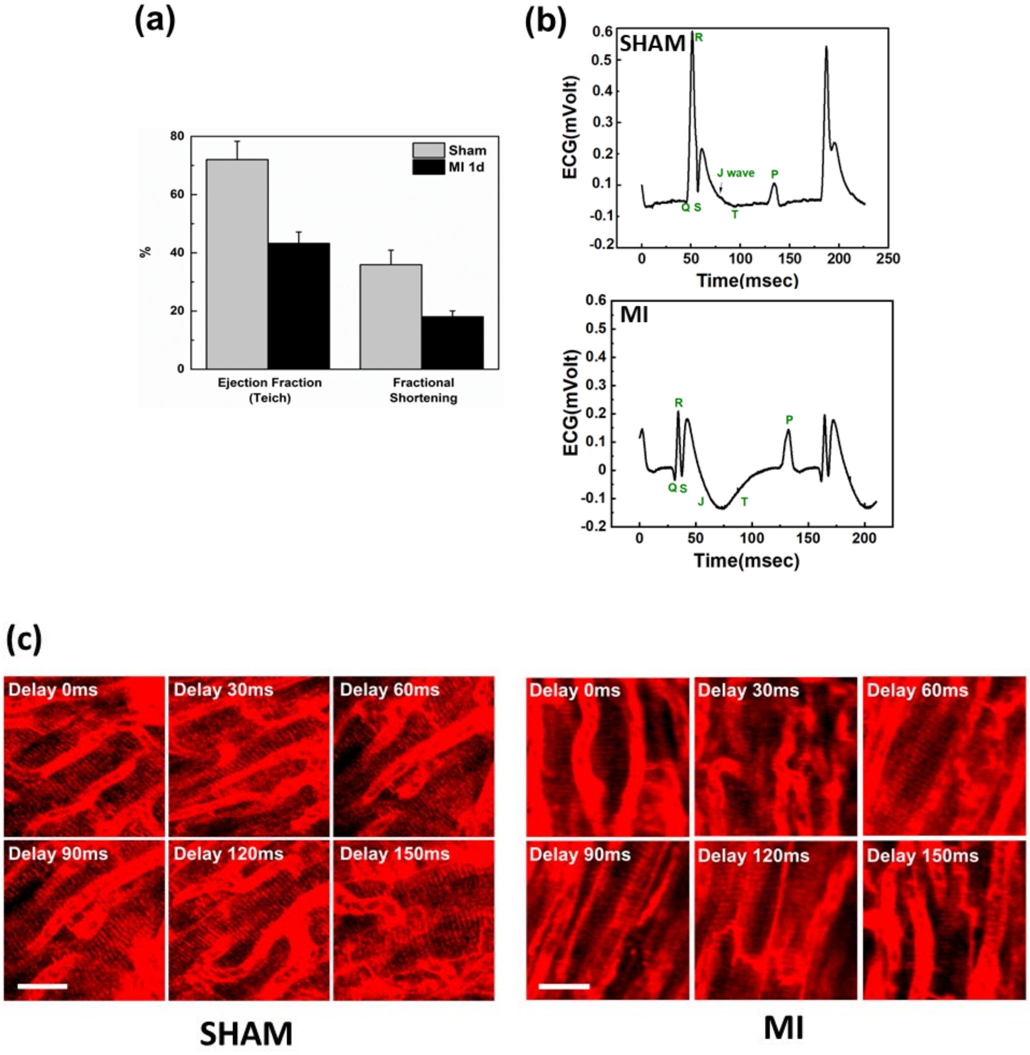

Supplementary Figure S6. (a) Left ventricular activity fraction, ejection fraction (EF %), and fraction shortening (FS %) in the sham and 1-day post-MI mice model (N sham=4, N MI= 10). (b) The ECG signal synchronized to sarcomere length displacement of healthy mice and MI –mice in Figure 3. (c) The images of cardiomyocytes at different phases of contraction cycle in both sham (right) and MI model (left). Scale bar:20 µm

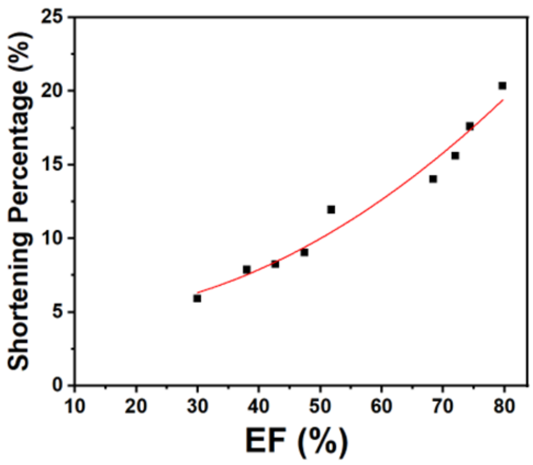

Supplementary Figure S7. Relationship between the sarcomere shortening percentage and ejection fraction (EF)

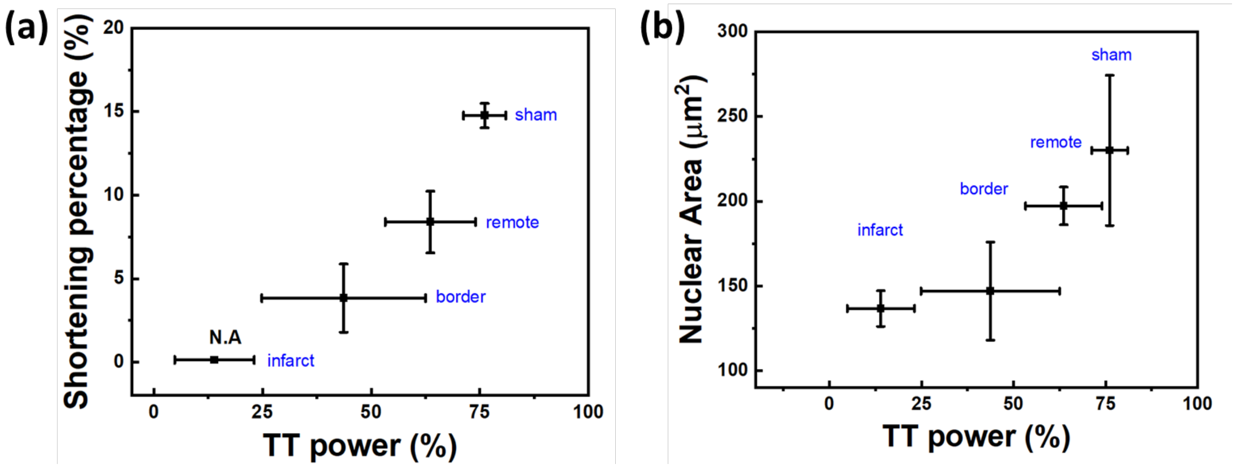

Supplementary Figure S8. Relationship between morphological change and regularity of sarcomere (TT power) with (a) shortening percentage and (b) the nucleus area.

Movies:

Movie S1: The time-lapse images of a beating heart in a mouse model were recorded by a conventional two-photon microscope without a stabilizer. The blur images were due to the motion of heartbeats and breathing. The di-2-anepeq dye (red) was used to label the cardiomyocytes.
Movie S2: The time-lapse images of a beating heart in a mouse model were recorded by a conventional two-photon microscope with a stabilizer. The acquisition rate is (a) 30 Hz and (b) 100 Hz
Movie S3: The time-lapse images of a beating heart in a mouse model were recorded by a conventional two-photon microscope with a stabilizer and synchronized with ECG signals. The images were recorded at different delay times (a) 0 ms, (b) 5ms, (c) 10 ms, (d) 20 ms with respect to the R peak of the ECG signals. It can be seen that clear images can be obtained at different contraction cycles at the same phase.
Movie S4: The recorded images of a beating heart with (a) the presence of only the motion compensation stabilizer and (b) the presence of both motion compensation stabilizer and ECG synchronization. Scale bar 200 µm
Movie S5: (a) Time-lapse image of cardiomyocytes in a beating heart labeled with di-2-anepeq dye (red) and Hoechst (blue), (b) The size variations of nuclei during contraction cycles, and (c) shape variations of the nucleus during the contraction cycles in the healthy mice.
Movie S6: (a) The size variations of nuclei during contraction cycles, and (b) shape variations of the nuclei during the contraction cycles in the MI model.
